# 2397: A checklist for developing and implementing a high-impact monitoring and evaluation system in clinical and translational science programs

**DOI:** 10.1017/cts.2017.269

**Published:** 2018-05-10

**Authors:** Boris Volkov

## Abstract

OBJECTIVES/SPECIFIC AIMS: This presentation will highlight the framework and domains of the monitoring and evaluation (M&E) System Checklist created in response to the need for practical guidelines and intended to improve the quality, efficiency, and consistency of monitoring and evaluation of the clinical and translational work. The recently published NCATS Strategic Plan (2016; p. 18) presents the following objectives and guidelines that implicitly suggest the need for sound M&E: “Objective 4-1: Continually assess and optimize internal business practices” and “Objective 4-2: Ensure all scientific programs and operational activities are conducted in a rigorous, robust and data-driven manner.” Given the complexity of clinical and translational work and associated monitoring/evaluation processes and the dearth of practical tools in the CTR evaluation area, the need for such a checklist is clear. A “checklist” (a detailed list of items/steps required, things to be done, or points to be considered) is a type of informational job aid used to improve performance, reduce failure, deal with complexity, and ensure consistency and completeness in carrying out work. Checklists are popular in many fields—due to their brevity, concreteness, order, implicit (and sometimes explicit) mandate to do things right, and expectation for a checklist’s being grounded in good practices and/or strong theory. A notable example is the famed WHO Surgical Safety Checklist (2008). The proposed M&E Checklist has been developed based on the author’s extensive experience in internal evaluation, checklist development and use, and working with the Clinical and Translational Sciences Awards (CTSAs)—as the UMN CTSI M&E Director, ACTS Evaluation SIG Chair, and a Co-Lead of the Evaluators Working Group within the NCATS CTSA Common Metrics Initiative. Although there is no “golden” algorithm that will totally suit every organization, the M&E checklist provides useful guidelines for building M&E. The Checklist presents the key concepts and important issues in M&E development and implementation. It also incorporates a synthesis of 3 grounded frameworks: King and Volkov’s Framework for Building Evaluation Capacity (2005), Simister’s Framework for Developing M&E Systems for Complex Organizations (2009), and the award-winning CDC Framework for Program Evaluation in Public Health (1999). For the purposes of the proposed Checklist, an M&E system (or framework/approach) is understood as “a series of policies, practices and processes that enable the systematic and effective collection, analysis and use of monitoring and evaluation information” (Simister, 2009; p. 1). A well-designed M&E system ensures a consistent approach to the collection, analysis, and use of information, while allowing considerable scope for different parts of an organization to develop and apply their own solutions in response to their particular situations. The M&E Checklist structured around 3 key domains (adapted from the Volkov and King ECB Checklist, 2007): (1) M&E/organizational context: taking advantage of the internal and external organizational context, administrative culture, and decision-making processes. (2) M&E structures: creating structures—mechanisms within the organization—that enable the M&E development and use. (3) M&E resources: making M&E resources available and used. For each domain, the Checklist has a number of associated categories and activities. Specifically, the checklist adopts and adapts the following useful steps from Simister’s approach: “Define the scope and purpose,” “Perform a situational analysis,” “Consult with relevant stakeholders,” “Identify the key levels and focus areas,” and “Integrate the M&E system horizontally and vertically,” as well as the CDC Framework’s steps “Engage stakeholders,” “Focus the M&E Design,” and “Ensure use and share lessons learned.”With slight modification, the organizations can also utilize the Checklist as a rubric/assessment tool to gauge the status of their M&E capacity. METHODS/STUDY POPULATION: A case study of methodological/implementation tool development. There are no human subjects in this study, thus, Study Population is not applicable to this study. This study is not subject to IRB review. RESULTS/ANTICIPATED RESULTS: The proposed checklist approach shows sound promise to not only impact individual programs and their M&E systems but to also enhance internal evaluation capacity, critical thinking, learning, strategic management, and improvement within clinical and translational science organizations. DISCUSSION/SIGNIFICANCE OF IMPACT: The ultimate goal and impact of the proposed checklist is to help ensure that organizations and their M&E teams consistently follow a few critical steps and thereby maximize the quality, efficiency, and consistency of monitoring and evaluation of the clinical and translational work. The checklist’s impact is significant in that it fills the current gap in the practice, literature, and methodology and provides practical guidance for CTR (and other) organizations and programs striving to improve the quantity and quality of evaluation.

**References**

**Centers for Disease Control and Prevention (CDC)**. *Framework for program evaluation in public health*. MMWR 1999; 48 (no. RR-11).

**King JA, Volkov B**. A framework for building evaluation capacity based on the experiences of three organizations. *CURA Reporter* 2005; **35**(3): 10–16.

**National Center for Advancing Translational Sciences**. *NCATS Strategic Plan* [Internet], 2016. NIH. (https://ncats.nih.gov/strategicplan)

**Simister N**. *Developing M&E systems for complex organisations: a methodology*. INTRAC, 2009.

**Volkov B, King J**. *A checklist for building organizational evaluation capacity* [Internt], 2007 (https://www.wmich.edu/sites/default/files/attachments/u350/2014/organiziationevalcapacity.pdf)

**World Alliance for Patient Safety**. *WHO surgical safety checklist and implementation manual* [Internet], 2008 (http://www.who.int/patientsafety/safesurgery/ss_checklist/en/)

